# Prevalent metabolic derangement and severe thrombocytopenia in ABO-incompatible liver recipients with pre-transplant plasma exchange

**DOI:** 10.1038/s41598-018-24887-x

**Published:** 2018-04-27

**Authors:** Hye-Mee Kwon, In-Gu Jun, JungBok Lee, Young-Jin Moon, Kyeo-Woon Jung, Hye-Won Jeong, Yong-Seok Park, Jun-Gol Song, Gyu-Sam Hwang

**Affiliations:** 1 Department of Anesthesiology and Pain Medicine, Laboratory for Cardiovascular Dynamics, Asan Medical Center, University of Ulsan College of Medicine, 88 Olympic-ro 43-gil, Songpa-gu, Seoul, 05505 Republic of Korea; 2 Department of Clinical Epidemiology and Biostatistics, Asan Medical Center, University of Ulsan College of Medicine Seoul, 88 Olympic-ro 43-gil, Songpa-gu, Seoul, 05505 Republic of Korea

## Abstract

Desensitisation with therapeutic plasma exchange (TPE) is essential for ABO-incompatible (ABO-I) liver transplants (LTs). However, excessive citrate load and coagulation disturbances after TPE have been poorly studied, in particular in cirrhotic patients with hypocapnic alkalosis, metabolic compensation and electrolyte imbalances. We retrospectively evaluated 1123 consecutive LT recipients (923 ABO-compatible [ABO-C], 200 ABO-I) from November 2008 to May 2015. TPE was generally performed a day before LT and blood sampling was performed before anaesthesia induction. We performed propensity score matching (PSM) and inverse probability treatment weighting (IPTW) analyses. In 199 PSM pairs, metabolic alkalosis was prevalent in ABO-I LT recipients (expectedly due to citrate conversion) with higher pH ≥ 7.50 (IPTW-adjusted odds ratio [aOR] = 2.23) than in ABO-C LT recipients. With increasing cirrhosis severity, the arterial pH and bicarbonate levels showed dose-dependent relationships, whereas mild hypoxaemia was more prevalent in ABO-I LT recipients. ABO-I LT recipients exhibited worsened hypokalaemia ≤3.0 mmol/l (17.6%, aOR = 1.44), hypomagnesaemia ≤1.7 mg/dl (27.6%, aOR = 3.43) and thrombocytopenia <30,000/µl (19.1%, aOR = 2.26) confirmed by lower maximal clot firmness (*P* = 0.001) in rotational thromboelastometry (EXTEM), which necessitated platelet transfusions. Preoperative identification of these change may prevent worsening of severe electrolyte disturbances and thrombocytopenia for optimal LT anaesthesia.

## Introduction

Liver cirrhosis (LC) has deleterious effects on many organ systems and is accompanied by metabolic and acid-base disturbances such as hyperventilatory hypocapnic alkalosis with metabolic compensation and electrolyte imbalances^1–3^. Therefore, understanding and managing such disturbances are important during the perioperative care of LC patients undergoing liver transplant (LT)^1,2,4–6^.

Living-donor LT (LDLT) is prevalent in Asian countries due to the limited deceased donor pools. Despite the initial poor outcomes, ABO-incompatible (ABO-I) LDLT emerged as a feasible method with significantly improved outcomes after implementing desensitisation protocols such as rituximab and preoperative therapeutic plasma exchange (TPE)^7–9^. Currently, ABO-I LDLT comprises approximately 20% of the total annual number of LDLT cases in Asian institutions^10^. In addition, recent studies are actively investigating the efficacy of TPE as a rescue and bridging treatment to LT for acute liver failure^11,12^.

Anticoagulation for TPE utilises citrate. Its safety and possible metabolic complications in patients with liver dysfunction have been a concern, and the fresh frozen plasma (FFP), used as replacement fluid as much as 1.5 times the plasma volume to reduce the isoagglutinin (IA) titre, may additionally augment the citrate level^7,8,13–15^. Massive amounts of citrate loading in the cirrhotic population has been an issue due to prolonged half-life of citrate^16^ and decreased bicarbonate consumption^17^. It may cause hypocalcaemia and hypomagnesaemia, inducing cardiac arrhythmias^16^, and cause pronounced acid-base imbalances, even after the cessation of TPE. Thus, severe metabolic alkalosis with subsequent electrolyte imbalance (such as hypokalaemia) may elicit respiratory derangements possibly due to compensatory hypoventilation^18–20^. Additionally, during TPE, platelets are inadvertently removed or their haemostatic capacity is impaired or hemodiluted^21,22^, which may have great impact since coagulation in cirrhotic patients is very dependent on the presence of sufficient platelets^23^. Therefore, re-evaluation of clinical conditions and laboratory markers immediately before LT, not a day before, should be emphasized in optimal management of ABO-I LT.

Few studies have demonstrated the feasibility of citrate anticoagulation in patients with cirrhosis^16^; however, a systematic assessment of complications and outcomes in this special cohort who undergo LT usually a day after TPE has been poorly studied. Given its reported decreased citrate metabolism in LT recipients, we set up to evaluate the hypotheses that pre-transplant TPE with citrate alters the expected pattern of the acid-base status, worsens respiration dysfunction, provokes electrolyte imbalances and aggravates the coagulation dysfunction. We also assessed whether the aggravated coagulation status has an impact on the need for intraoperative transfusions.

## Results

### Study population

All ABO-I LDLT recipients received TPE prior to surgery. The median number of TPE sessions was 4 (range, 1–14). Eight patients (4%) underwent their last TPE on the morning of the surgery, 182 patients (91%) on the day before surgery, nine patients (4.5%) two days before surgery and one patient (0.5%) six days before surgery. The mean estimated plasma volume removed from the patient and replaced with a similar volume of FFP was 2793 ml (range, 1539–4096 ml). None of the ABO-C LDLT recipients received TPE.

The characteristics of the 923 ABO-C and 200 ABO-I LDLT recipients receiving TPE are shown in Table 1. After propensity score (PS) matching (PSM), model discrimination and calibration statistics showed good c-statistics (c = 0.7406) and Hosmer-Lemeshow statistics (chi-square = 3.9837, degrees of freedom = 8, P = 0.8586). Under the statistical PSM option, 199 pairs were chosen, and the imbalances between the baseline covariates were diminished, as shown by the standardised mean difference <0.2 (mostly ≤ 0.1) (Table 2).

**Table 1:** Baseline characteristics and intraoperative variables of ABO-C and ABO-I liver transplant recipients and donor characteristics.

	ABO-C (n = 923)	ABO-I (n = 200)	Total (n = 1123)	*P* value
**Patient demographics**				
Sex, male, %	721 (78.1)	146 (73.0)	867 (77.2)	0.142
Age, years	53 (49–58)	54 (49–57)	53.0 (49.0–58.0)	0.952
Body mass index, kg/m^2^	23.9 ± 3.2	23.6 ± 3.1	23.9 ± 3.1	0.205
MELD score	13.0 (9.0–18.0)	11.5 (8.0–14.5)	12.0 (9.0–17.0)	<0.001
MELD score per tertile ≤10/11–20/>20), %	353 (38.2)/387 (41.9)/ 183 (19.8)	92 (46.0)/97 (48.5)/ 11 (5.5)	445 (39.6)/484 (43.1)/ 194 (17.3)	<0.001
Child-Turcotte-Pugh classification (A/B/C)	304 (32.9)/341 (36.9)/ 278 (30.1)	71 (35.5)/104 (52.0)/ 25 (12.5)	375(33.4)/445(39.6)/ 303(27.0)	<0.001
Mean arterial pressure, mmHg	81 (76–88)	80 (74–86)	81.0 (76.0–88.0)	0.018
SpO2, %	97 (96–98)	97 (96–98)	97.0 (96.0–98.0)	0.926
Ventilatory care, %	21 (2.3)	1 (0.5)	22 (2.0)	0.174
Preoperative vasopressor use, %	5 (0.5)	0 (0.0)	5 (0.4)	0.647
Pre-TPE platelet transfusion, %	—	17 (8.5)	—	—
Pre-LT hospital stay, days	5 (2–9)	8 (7–15.5)	6.0 (3.0–10.0)	<0.001
Prior abdominal operation history, %	97 (10.5)	17 (8.5)	114 (10.2)	0.049
**Comorbidities**				
Diabetes, %	215 (23.3)	21 (10.5)	236 (21.0)	<0.001
Hypertension, %	145 (15.7)	24 (12.0)	169 (15.0)	0.220
Coronary artery disease, %	37 (4.0)	13 (6.5)	50 (4.5)	0.174
Hepatic encephalopathy, %	132 (14.3)	19 (9.5)	151 (13.4)	0.091
Hydrothorax, %	137 (14.8)	22 (11.0)	159 (14.2)	0.193
Refractory ascites, %	69 (7.5)	21 (10.5)	90 (8.0)	0.199
Oesophageal varix, %	241 (26.1)	63 (31.5)	304 (27.1)	0.142
Ascites, grades	2 (1–3)	2 (1–3)	2.0 (1.0–3.0)	0.385
Current use of beta blocker, %	202 (21.9)	46 (23.0)	248 (22.1)	0.802
Current use of diuretic, %	331 (35.9)	87 (43.5)	418 (37.2)	0.052
**Preoperative variables**				
Total bilirubin, mg/dl	1.9 (1.1–4.7)	1.8 (1.0–2.8)	1.9 (1.1–4.2)	0.007
Creatinine, mg/dl	0.74 (0.62–0.87)	0.69 (0.57–0.82)	0.73 (0.61–0.87)	0.004
Estimated GFR^*^, ml/min/1.73m^2^	60 (60–89)	60 (60–80.5)	60 (60–89)	0.003
Aspartate aminotransferase >40 IU/l, %	522 (56.6)	56 (28.0)	578 (51.5)	<0.001
Alanine aminotransferase >40 IU/l, %	250 (27.1)	19 (9.5)	269 (24.0)	<0.001
Estimated plasma volume, ml	—	2793 (2522–3053)	—	—
**Original liver disease, %**				
Hepatitis B virus	625 (67.7)	149 (74.5)	774 (68.9)	0.073
Hepatitis C virus	77 (8.3)	16 (8)	93 (8.3)	0.986
Alcoholic cirrhosis	143 (15.5)	24 (12)	167 (14.9)	0.251
Other diseases	102 (11.1)	20 (10.0)	122 (10.9)	0.758
Combined HCC	508 (55.0)	110 (55.0)	618 (55.0)	1.000
**Donor- and operation-related variables**				
Donor age, years; n = 1078	26.0 (21.0–32.0)	27.0 (22.0–32.0)	26.0 (21.0–32.0)	0.334
Donor sex, male; n = 1078	639 (72.0)	137 (72.1)	776 (72.0)	1.000
Donor body mass index, kg/m^2^; n = 1078	22.6 (20.6–24.6)	23.4 (21.6–25.4)	22.7 (20.8–24.7)	0.001
Graft steatosis, %; n = 1074	1.0 (0.0–5.0)	1.0 (0.0–5.0)	1.0 (0.0–5.0)	0.827
Cold ischaemic time, mins; n = 1074	80 (66–97)	83 (69–97.5)	81 (66.0–97.0)	0.153
Warm ischaemic time, mins; n = 1074	40 (33–50)	39 (33.5–48)	40.0 (33.0–50.0)	0.549

^*^Values are expressed as mean (±standard deviation) or median and interquartile range for continuous variables as appropriate, and n (%) for categorical variables.

Estimated GFR^*^, Calculated by six-variable Modification of Diet in Renal Disease.

ABO-C, ABO-compatible; ABO-I, ABO-incompatible; GFR, glomerular filtration rate; HCC, hepatocellular carcinoma; LT, liver transplantation; MELD, Model for End-Stage Liver Disease; SpO_2_, oxygen saturation; TPE, therapeutic plasma exchange.

**Table 2:** Baseline characteristics of ABO-C and ABO-I liver transplant recipients after propensity score matching analysis.

	Propensity-matched set			
	ABO-C (n = 199)	ABO-I (n = 199)	*P* value	Standardised mean difference
**Patient demographics**				
Sex, male, %	143 (71.9)	146 (73.4)	0.822	0.034
Age, years	52 (48–58)	54 (49–57)	0.359	0.045
Body mass index, kg/m^2^	23.4 ± 3.2	23.6 ± 3.1	0.470	0.072
MELD score	11.0 (8.0–15.5)	11.0 (8.0–14.5)	0.726	0.089
MELD ≥ 15, %	60 (30.2)	50 (25.1)	0.313	0.113
Ventilatory care, %	2 (1.0)	1 (0.5)	1.000	0.058
**Comorbidities**				
Diabetes, %	22 (11.1)	21 (10.6)	1.000	0.016
Hypertension, %	19 (9.6)	24 (12.1)	0.518	0.081
Coronary artery disease, %	16 (8.0)	12 (6.0)	0.557	0.079
Hepatic encephalopathy, %	21 (10.6)	19 (9.5)	0.868	0.033
Hydrothorax, %	22 (11.1)	22 (11.1)	1.000	<0.001
Refractory ascites, %	18 (9.0)	20 (10.1)	0.865	0.034
Oesophageal varix, %	54 (27.1)	62 (31.2)	0.440	0.089
Ascites, grades	2 (1–3)	2 (1–3)	0.369	0.100
Current use of beta blocker, %	48 (24.1)	46 (23.1)	0.906	0.024
Current use of diuretic, %	95 (47.7)	86 (43.2)	0.421	0.091
**Preoperative variables**				
Total bilirubin, mg/dl	1.4 (0.9–2.9)	1.8 (1.0–2.8)	0.208	0.004
Creatinine, mg/dl	0.71 (0.60–0.84)	0.70 (0.57–0.82)	0.453	0.014
Aspartate aminotransferase >40 IU/l, %	59 (29.7)	55 (27.6)	0.739	0.044
Alanine aminotransferase >40 IU/l, %	18 (9.1)	18 (9.1)	1.000	<0.001
Estimated plasma volume, ml	—	2793 (2526–3053)	—	—
**Original liver disease, %**				
Hepatitis B virus	141 (70.9)	140 (70.4)	1.000	0.011
Hepatitis C virus	14 (7.0)	15 (7.5)	1.000	0.019
Alcoholic cirrhosis	27 (13.6)	24 (12.1)	0.764	0.045
Other diseases	17 (8.5)	20 (10.1)	0.730	0.052
Combined HCC	108 (54.3)	110 (55.3)	0.920	0.020

^*^Values are expressed as mean (±standard deviation) or median and interquartile range for continuous variables, as appropriate, and n (%) for categorical variables.

ABO-C, ABO-compatible; ABO-I, ABO-incompatible; HCC, Hepatocellular carcinoma; MELD, Model for End-Stage Liver Disease.

### Evaluation of acid-base status and electrolyte disturbances

**Figure 1:**
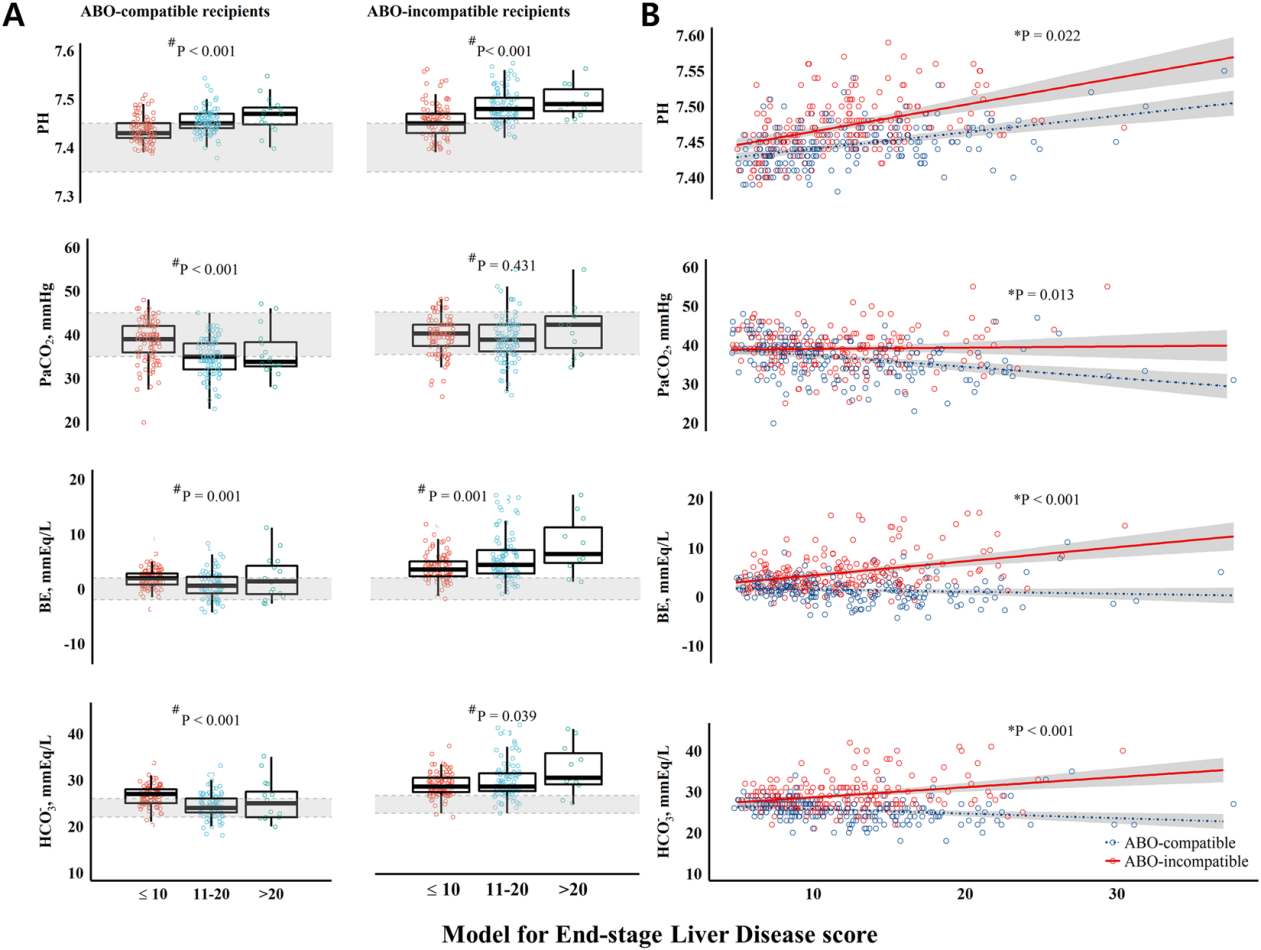
(**A**) Box and dot plot in the left panel and (**B**) dot plot with fitted line in the right panel show differences in pH, PaCO_2_, BE and HCO_3_^−^ between ABO-compatible and ABO-incompatible liver transplant recipients according to the Model for End-Stage Liver Disease score after propensity score matching analysis. Shaded areas in the left panel show the normal range, whereas those in the right panel depict 95% confidence intervals. ^#^P value showing difference between the Model for End-Stage Liver Disease scores tertiles (Jonckheere-Terpstra test). *P value showing difference between ABO-compatible and ABO-incompatible liver transplant recipients (analysis of covariance test). BE, base excess; PaCO_2_, partial pressure of carbon dioxide.

**Figure 2:**
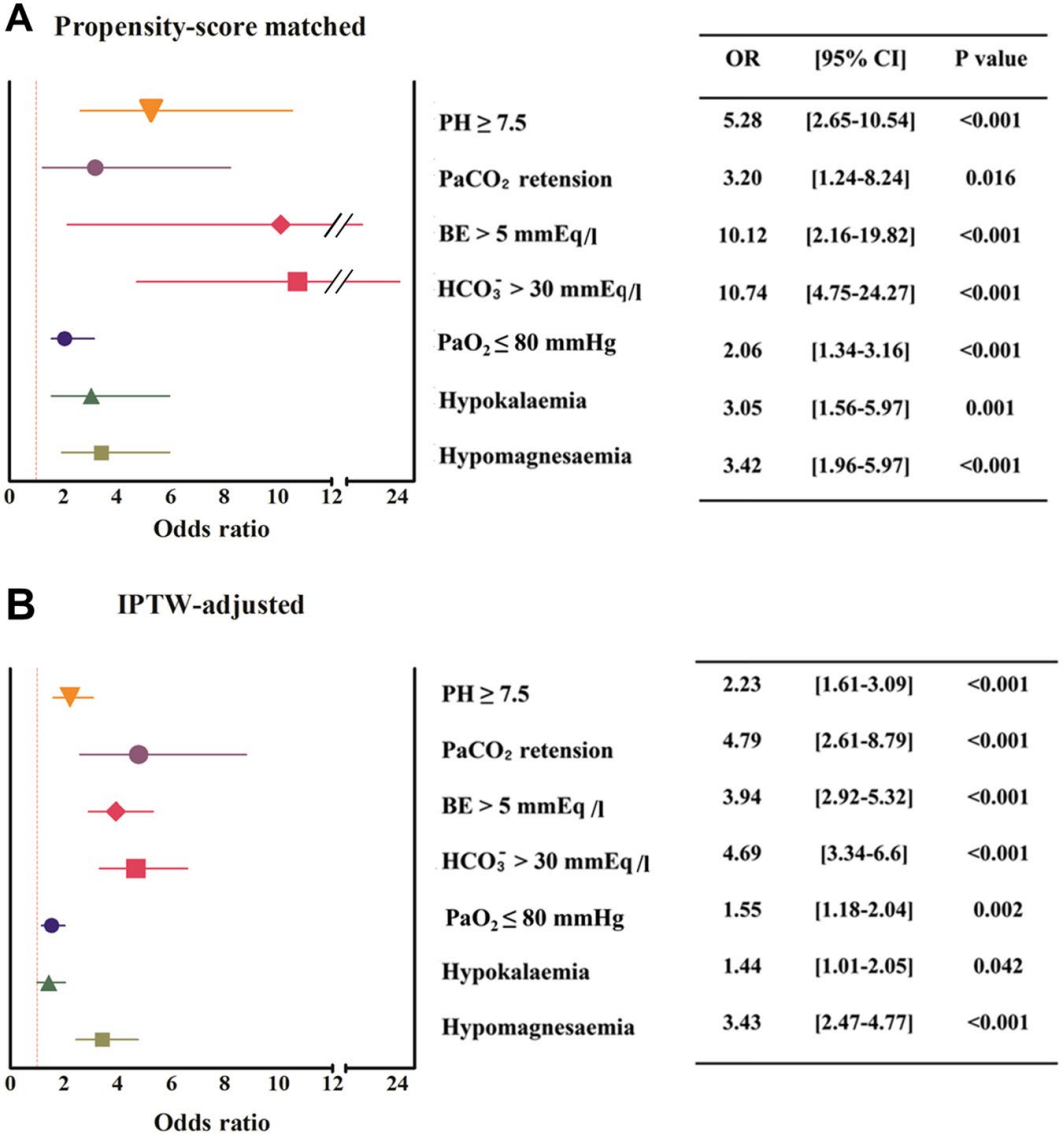
(**A**) Propensity score-matched odds ratio and (**B**) inverse probability of treatment weighting (IPTW)-adjusted odds ratio of laboratory findings. BE, base excess; CI, confidence interval; IPTW, inverse probability of treatment weighting; PaCO_2_, partial pressure of carbon dioxide; PaO_2_, partial pressure of oxygen; OR, odds ratio.

The acid-base statuses and electrolyte disturbances evaluated immediately before anaesthesia induction are shown in Figure 1 and Table 3. The arterial pH increased in a dose-dependent fashion with LC severity in ABO-C LDLT recipients. While matched ABO-C LDLT recipients (n = 199) showed hypocapnic respiratory alkalosis, the ABO-I LDLT recipients presented an altered acid-base balance, showing metabolic alkalosis with increased base excess (BE) and HCO_3_^−^ (Fig. 1). Also, compared with matched ABO-C, ABO-I LDLT recipients showed a higher rate of severe alkalosis (5.5% *vs*. 23.6%, *P* < 0.001). Figure 2 shows both the PSM odds ratio (OR) and the inverse probability of treatment weighting (IPTW)-adjusted odds ratio (aOR). ABO-I LDLT recipients had a higher rate of severe alkalosis (aOR 2.23, P < 0.001) than ABO-C LDLT recipients. Compared to pre-TPE, the severe alkalosis significantly developed after TPE in ABO-I LDLT recipients (n = 94, 6.4% *vs*. 25.5%, P < 0.001) (Supplementary Table S1).

**Table 3:** Acid-base balance, electrolytes, respiratory analyses, blood counts, and coagulation parameters evaluated immediately before initiating anesthesia and intraoperative transfusions in ABO-C and ABO-I liver transplant recipients after propensity score matching analysis.

	ABO-C (*n* = 199)	ABO-I (*n* = 199)	*P* value
**Acid-base balance**			
pH	7.45 ± 0.03	7.47 ± 0.04	<0.001
pH ≥ 7.50, %	11 (5.5)	47 (23.6)	<0.001
Base excess, mmEq/l	1.39 ± 2.39	4.97 ± 3.76	<0.001
HCO_3_^−^, mmEq/l	25.5 ± 2.8	29.1 ± 3.8	<0.001
HCO_3_^−^ > 30 mmEq/l, %	7 (3.5)	56 (28.1)	<0.001
PaCO_2_, mmHg	37 ± 4.96	39 ± 4.95	<0.001
PaCO_2_ > 45 mmHg, %	6 (3.0)	18 (9.0)	0.021
PaO_2,_ mmHg	88.0 ± 10.6	84.3 ± 11.2	0.001
PaO_2_ ≤ 80 mmHg	49 (24.6)	80 (40.2)	0.001
Lactic acid, mmol/l	0.99 ± 0.37	1.07 ± 0.36	0.004
**Electrolyte analysis**			
Sodium, mmol/l	136 ± 4.13	138 ± 3.4	0.001
Potassium, mmol/l	3.7 ± 0.43	3.4 ± 0.45	<0.001
Potassium ≤ 3 mmol/l, %	13 (6.5)	35 (17.6)	0.001
Magnesium, mg/dl	1.98 ± 0.25	1.86 ± 0.23	<0.001
Magnesium ≤ 1.7 mg/dl, %	20 (10.1)	55 (27.6)	<0.001
Total calcium, mmol/l	2.03 ± 0.15	2.07 ± 0.18	0.011
Ionised calcium, mmol/l	1.11 ± 0.07	1.12 ± 0.07	0.443
Ratio of total to ionised calcium >2.1, %	7 (3.5)	18 (9.0)	0.039
**Coagulation parameters**			
Platelets, ×10^3^ /µl	64 (49–91)	47 (32–72)	<0.001
Platelets <30 × 10^3^ /µl	12 (6.0)	38 (19.1)	<0.001
Fibrinogen, mg/dl	173 (131–226)	165 (145–193)	0.256
Antithrombin III,%	52 (34–70)	61 (50–75)	<0.001
PT, INR	1.33 (1.14–1.55)	1.25 (1.14–1.42)	0.018
aPTT, s	33 (29–37)	31 (28–34)	<0.001
**Transfusion**			
Packed red blood cell transfusion, units	6.0 (2.0–14.0)	7.0 (3.0–12.0)	0.910
Fresh frozen plasma transfusion, units	8.0 (3.0–14.5)	7.0 (2.0–12.0)	0.165
Cryoprecipitate transfusion, units	10.0 (0–10)	10.0 (0–10)	0.207
Apheresis platelet transfusion, %	102 (51.3)	126 (63.3)	0.020

^*^Values are expressed as mean (±standard deviation) or median and interquartile range as appropriate, and n (%) for categorical variables. ABO-C, ABO-compatible; ABO-I, ABO-incompatible; aPTT, activated partial thromboplastin time; INR, international normalised ratio; PT, prothrombin time.

In accordance with the pH values, ABO-I LDLT recipients showed a significantly higher level of BE (4.97 ± 3.76 mmEq/l *vs.*1.39 ± 2.39 mmEq/l, P < 0.001) and HCO_3_^−^ (29.1 ± 3.8 mmEq/l *vs.* 25.5 ± 2.8 mmEq/l, P < 0.001) and a higher rate of HCO_3_^−^ > 30 mmEq/l (28.1% *vs.* 3.5% *vs*., P < 0.001) than the ABO-C LDLT recipients after PSM. Identical findings were seen when comparing measurements before and after TPE; BE changed from −2.22 ± 3.04 mmEq/l to 5.13 ± 3.86 mmEq/l and HCO_3_^−^ increased from 22.0 ± 3.0 mmEq/l to 29.2 ± 3.9 mmEq/l after TPE (Supplementary Table S1). This suggests that the ability to metabolise citrate is preserved to a certain degree in LT recipients. Therefore, the pH difference is largely due to the exogenous bicarbonate converted from the citrate load used in TPE. Additionally, we observed worsened pre-existing electrolyte imbalances as a consequence of the metabolic alkalosis. Higher rate of severe hypokalaemia was observed in ABO-I LDLT recipients, compared to ABO-C LDLT recipients after PSM analysis (≤3.0 mmol/l, 17.6% *vs*. 6.5%, P = 0.001). Moreover, a higher rate of hypomagnesaemia—a sign of excess citrate—was seen in ABO-I LDLT recipients (27.6% *vs*. 10.1%, P < 0.001).

### Respiratory disturbance

**Figure 3:**
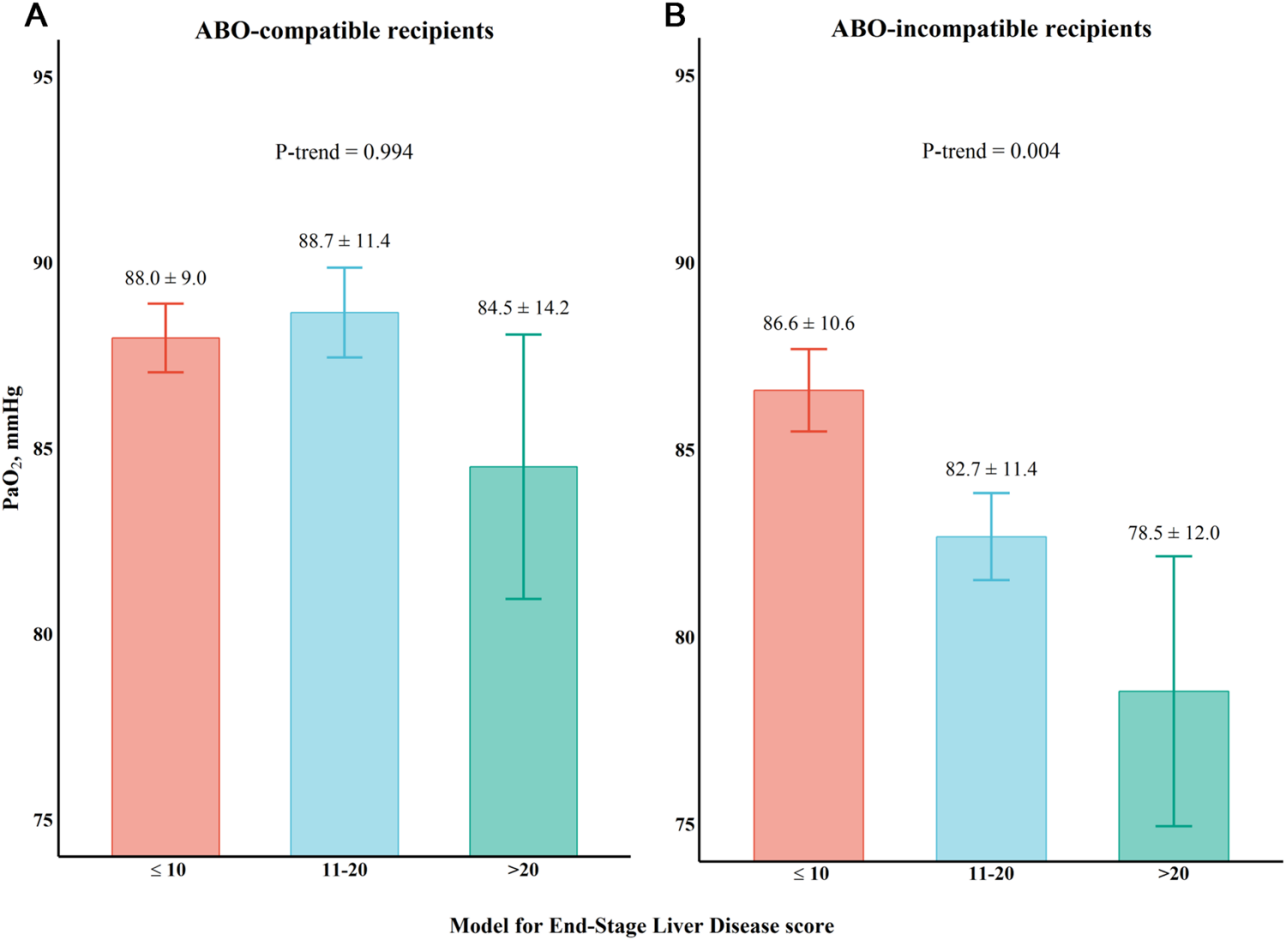
Differences in PaO_2_ between (**A**) ABO-C and (**B**) ABO-I liver transplant recipients according to the Model for End-Stage Liver Disease score tertiles (≤10, 11–20, >20) after propensity score matching analysis. A clear dose-dependent relationship of PaO_2_ with the Model for End-Stage Liver Disease score in ABO-I patients is demonstrated in the right panel (**B**). ABO-C, ABO-compatible; ABO-I, ABO-incompatible; PaO_2_, partial pressure of oxygen.

ABO-I LDLT recipients showed a significantly higher partial pressure of carbon dioxide (PaCO_2_) level (39 ± 4.95 mmHg *vs*. 37 ± 4.96 mmHg, P < 0.001) and a higher rate of PaCO_2_ retention (PaCO_2_ ≥ 45 mmHg; 9.0% *vs*. 3.0%, P = 0.021) than ABO-C LDLT recipients. More importantly, they showed a lower partial pressure of oxygen (PaO_2_) level (P = 0.001) (Fig. 3, Table 3) and a higher rate of PaO_2_ ≤ 80 mmHg (40.2% *vs*. 24.6%, P = 0.001) after PSM. Compared with matched ABO-C LDLT recipients, the lower PaO_2_ (P = 0.004) in ABO-I LDLT recipients’ tertiles exhibited a dose-dependent relationship with the LC severity (31.5% *vs*. 45.8% *vs*. 63.6%, from low to high LC severity, P = 0.036) (Fig. 3). The ORs of hypercapnia and PaO_2_ ≤ 80 mmHg are shown in Figure 2.

### Evaluation of coagulation disturbances and intraoperative transfusion

The conventional coagulation test results in Table 3 show significantly lower mean platelet counts (47,000/µl *vs*. 64,000/µl, P < 0.001) in ABO-I compared with ABO-C LDLT recipients. Of note, the mean platelet counts before TPE were 58,000/µl, but they were significantly lowered after TPE (P < 0.001). We observed a significantly higher rate of severe thrombocytopenia (<30,000/µl, 19% *vs*. 6%, P < 0.001) in ABO-I LDLT recipients, which resulted in more platelet transfusions during LT surgery. The risk of severe thrombocytopenia was significantly higher in ABO-I LDLT recipients (aOR 2.26, P < 0.001).

With a separate PSM analysis of ABO-I LDLT recipients who underwent rotational thromboelastometry (ROTEM; TEM International GmbH, Munich, Germany) analysis (*n* = 129, Baseline characteristics are shown in Supplementary Table S2), EXTEM and INTEM analyses revealed no significant differences in CT and CFT values, whereas the prothrombin time and activated partial thromboplastin time were improved. We found no statistical differences in either fibrinogen level, A10 or MCF in FIBTEM. However, A10 and MCF were significantly lower in ABO-I patients in both EXTEM and INTEM analyses as is expected by thrombocytopenia (Table 4).

**Table 4:** Preoperative rotational thromboelastometry analyses of ABO-C and ABO-I liver transplant recipients after propensity score matching analysis.

	Normal Range	ABO-C (*n* = 129)	ABO-I (*n* = 129)	*P* value
**INTEM**				
CT, s	100–240	201.5 ± 40.1	204.2 ± 87.7	0.756
CFT, s	30–110	225.7 ± 206.6	262.0 ± 150.8	0.108
A10, mm	44–66	36.7 ± 9.8	33.1 ± 8.3	0.002
MCF, mm	50–72	44.8 ± 10.8	41.1 ± 8.1	0.002
**EXTEM**				
CT, s	38–79	63.2 ± 36.0	59.4 ± 27.3	0.344
CFT, s	34–159	238.2 ± 233.4	280.7 ± 180.4	0.103
A10, mm	43–65	37.7 ± 10.4	33.0 ± 8.2	<0.001
MCF, mm	50–72	45.8 ± 11.4	41.6 ± 8.6	0.001
**FIBTEM**				
A10, mm	7–23	9.2 ± 3.7	8.5 ± 2.3	0.065
MCF, mm	9–25	9.9 ± 4.1	9.2 ± 2.6	0.091

PS model was discriminated with c-statistics (C = 0.6861) and model calibration was performed with Hosmer-Lemeshow statistics (χ2 = 8.0307, Degrees of freedom = 8, p = 0.431). *Values are expressed as mean ± standard deviation or median (interquartile range) for continuous variables, as appropriate, and n (%) for categorical variables. A10, clot amplitude at 10 minutes; ABO-C, ABO-compatible; ABO-I, ABO-incompatible; CFT, clot formation time; CT, clot time; MCF, maximum clot firmness.

The amounts of intraoperative transfusions are shown in Table 3. The percentage of patients who received apheresis platelets was significantly higher in the ABO-I group (63.3% *vs*. 51.3%, P = 0.02). This finding was confirmed with the OR (1.64, P = 0.015) and aOR (1.43, P = 0.01). However, no differences were found in the numbers of transfusion of packed red blood cells (pRBCs, P = 0.91), FFP (P = 0.165) or cryoprecipitates (P = 0.207).

### Evaluation of citrate metabolism

None of the ABO-I LDLT recipients had total calcium to ionized calcium ratio (tCa-to-iCa) values > 2.5, which is the best cut-off to detect citrate accumulation, and the lactic acid level was within the clinically normal range in both groups, showing no signs of citrate accumulation even in those with high MELD scores. However, the prevalence of tCa-to-iCa values > 2.1, which reflects a citrate overdose but not an accumulation, was higher in the ABO-I LT group (9.0% *vs*. 3.5%, PSM-P = 0.039).

### Clinical outcomes

The incidence of acute kidney injury (AKI) was significantly higher in ABO-I LDLT recipients (69% *vs*. 54%, PSM-P = 0.004) compared to ABO-C. Logistic regression after PSM showed that ABO-I was an independent risk factor for AKI (PSM-OR 1.88, P = 0.003). However, the intensive care unit stays were not different between ABO-C and ABO-I groups, before and after matching (All P > 0.05). During the median 4.78 years of follow-up, 30-day, 6-month and overall mortality and graft failure were not significantly different between the groups (All PSM-P > 0.05) as seen in Table 5.

**Table 5:** Clinical outcomes after liver transplantation between ABO-C and ABO-I liver transplant recipients.

	Crude				PS-matched set			
	Event (%)	OR	95% CI	*P* value	Event (%)	OR	95% CI	*P* value
Intensive care unit ≥7 days^*^								
ABO-C	129 (14.0)	1			28 (14.1)	1		
ABO-I	30 (15.0)	1.09	0.71–1.67	0.707	30 (15.1)	1.08	0.62–1.89	0.776
Acute kidney injury^‡^								
ABO-C	539 (58.4)	1			108 (54.3)	1		
ABO-I	137 (68.5)	1.55	1.12–2.15	0.008	137 (68.8)	1.88	1.24–2.85	0.003
	**Event (%)**	**HR**	**95% CI**	***P*** **value**	**Event (%)**	**HR**	**95% CI**	***P*** **value**
30-day mortality								
ABO-C	13 (1.4)	1			3 (1.5)	1		
ABO-I	2 (1.0)	0.71	0.16–3.13	0.647	2 (1.0)	0.66	0.11–3.97	0.654
6-month mortality								
ABO-C	37 (4.0)	1			7 (3.5)	1		
ABO-I	4 (2.0)	0.49	0.18–1.38	0.179	4 (2.0)	0.57	0.17–1.93	0.364
Overall mortality								
ABO-C	90 (9.8)	1			15 (7.5)	1		
ABO-I	9 (4.5)	0.46	0.23–0.91	0.026	9 (4.5)	0.61	0.27–1.39	0.236
Overall graft failure								
ABO-C	95 (10.3)	1			17 (8.5)	1		
ABO-I	14 (7.0)	0.69	0.39–1.21	0.194	14 (7.0)	0.85	0.42–1.73	0.663

ABO-C, ABO-compatible; ABO-I, ABO-incompatible; CI, confidence interval; HR, hazard ratio; OR, odds ratio; PS, propensity score.

Acute kidney injury^‡^: Classified according to Kidney Disease: Improving Global Outcomes.

Intensive care unit ≥7 days^*^: Compared to intensive care unit <7 days.

## Discussion

The first primary finding of this study is that the pre-transplant TPE, typically performed until the day before the LT surgery, regional citrate anticoagulation (RCA) and FFP altered acid-base status in LC patients, shifting it from respiratory alkalosis to the metabolic alkalosis. Consequently, the pre-existing electrolyte disturbances were worsened, and the risk of severe hypokalaemia and hypomagnesaemia were significantly higher in ABO-I LDLT recipients. Furthermore, metabolic alkalosis associated with TPE was responsible for worse respiratory profiles. An increased rate of PaO_2_ ≤ 80 mmHg was found with PaCO_2_ retention after TPE, possibly due to compensatory respiratory depression. Such findings are highly relevant for anaesthesiologists before anaesthesia induction and mechanical ventilation because the inadvertent hyperventilation, which is common in this period, may lead to mixed and severe alkalosis, due to the combination of respiratory alkalosis in response to a rapid fall in PaCO_2_ and the metabolic alkalosis by TPE. Additionally, anaesthesiologists need to be aware of the risk of a severe alkalosis associated with cardiocirculatory effects. Second, as a consequence of liver dysfunction, pH, HCO_3_^−^ and PaCO_2_ increased and PaO_2_ decreased with increasing LC severity in a dose-dependent manner. Third, thrombocytopenia was aggravated after TPE, which was evident in ROTEM analysis, and severe thrombocytopenia was more prevalent. These factors resulted in increased intraoperative platelet transfusion.

In an effort to expand the donor pool to meet the increasing organ demands, the barrier of ABO-I has been challenged with diverse desensitisation methods such as TPE, local graft infusion therapy, cyclophosphamide, splenectomy and intravenous immunoglobulin therapies. Currently, improved outcomes with desensitisation protocol of TPE with rituximab have allowed ABO-I LDLT to become a feasible option. As for anticoagulation during TPE, RCA is preferred over heparin in those with impaired coagulation because it does not cause heparin-induced thrombocytopenia, heparin resistance or risk of bleeding. Since patients with cirrhosis already have impaired coagulation with thrombocytopenia, these complications may be fatal in LT recipients. Citrate provides anticoagulation by chelating ionised calcium; thereafter, it is predominantly converted to bicarbonate in the liver and bicarbonate is excreted mainly through the kidney^24^. Accordingly, RCA in patients with hepatic failure has been the topic of interest in several clinical studies due to citrate accumulation concerns^16,20,25–27^. The citrate level can be inferred via an increased tCa-to-iCa or through the lactate level^20,25,26^. The tCa-to-iCa > 2.1 is the best cut-off to detect citrate overdose^21^ and a tCa-to-iCa > 2.5 reflects citrate accumulation, which is closely related to poor clinical outcomes^20,25,26^. In our results, none of the ABO-I LT recipients had tCa-to-iCa values > 2.5, and only some had a slightly increased rate of tCa-to-iCa values > 2.1. Lactic acid, another surrogate for citrate accumulation^26^, was also within the normal range in both groups. These results suggest that the hepatic handling capacity for citrate is preserved to certain degree in these LC patients, as reported^26,27^ and that citrate accumulation is not an issue regardless of LC severity in the current LC cohort. Rather, metabolic alkalosis from prolonged citrate conversion, with a higher rate of PaCO_2_ retention, lower PaO_2_ and aggravated hypokalaemia and hypomagnesaemia were more important findings before anaesthesia induction.

Metabolic alkalosis is a complication of excess citrate due to RCA with additional citrate loads from FFP, but only a few studies^28^ have concentrated on the metabolic complications of pre-transplant TPE in patients with LC undergoing LT immediately after TPE. Interestingly, current study shows that arterial pH, HCO_3_^−^ and respiratory derangement increased proportionally with LC severity. It should be pointed out that the liver plays an important role in regulating the acid-base balance through HCO_3_^−^ consumption^17^, and although progressive loss of hepatic urea synthesis occurs in stable LC patients, adequate level of HCO_3_^−^ disposal is maintained by the activation of liver glutaminases. However, an abrupt increase in HCO_3_^−^ converted from exogenous citrate could deteriorate this fragile balance, which may be a plausible explanation for the relationship between LC severity and acid-base imbalance.

For the optimal management of patients who undergo TPEs for ABO-I LT surgeries, anaesthesiologists need to be aware of such an altered preoperative acid-base status. For every 10 mmHg decrease in PaCO_2_, there is a concomitant 0.5 mmol/l decrease in potassium; therefore, consequent pH changes due to an acute decrease in the PaCO_2_ level can provoke sudden and significant hypokalaemia^29^. If one is unaware of the altered metabolic state after TPE, hypokalaemia may be unintentionally exacerbated by hyperventilation during anaesthesia induction in ABO-I LDLT recipients who already have a higher rate of severe hypokalaemia^29^. The adverse effects of hypokalaemia are numerous and include muscle weakness, ventilatory depression and, most importantly, a lowered threshold for cardiac arrhythmias^30^. About 50% of LC patients, who already have a reduced threshold for arrhythmia due to prevalent QTc prolongation, might have that risk increased by electrolyte imbalances^31,32^. In addition, our results revealed a higher rate of hypomagnesaemia in ABO-I LDLT recipients. Close monitoring and tight control of both hypokalaemia and hypomagnesaemia with appropriate management may be mandatory for preventing fatal arrhythmias in ABO-I LDLT recipients, given the possibility of torsades de pointes associated with hypotension and electrolyte disturbances in cirrhotic patients with severe QTc prolongation^33^ and with progressively prolonged QTc intervals during the pre-anhepatic and anhepatic phases^31,34^.

Attention should be paid to the preoperative oxygenation to make differential diagnosis of hypoxaemia in LC patients. Cirrhosis affects the respiratory function; ascites, atelectasis and pleural effusions are known to causes mild hypoxaemia, which is associated with LC severity^35^. Metabolic alkalosis is well known to decrease ventilation as respiratory compensation, resulting in hypercapnia and hypoxaemia^36–38^. Additionally, diminished ventilatory response to hypoxaemia and CO_2_ may aggravate the hypoxaemia^36,38^, with a leftward shift of the haemoglobin dissociation curve^39^. Our results emphasise the impact of an altered metabolic profile on physiologic variables and propose measures for safe anaesthesia induction and maintenance. Moreover, TPE exposes patients to large volume of exogenous plasma with related antigens and to risks of the extracorporeal circulation including the production of microthrombi entering the vascular bed and minor thromboses at the vascular access site. Patients with infectious conditions such as acute on chronic liver cirrhosis can be more vulnerable to external antigens, and complications such as transfusion-related lung injury are still reported despite minimisation strategies. Previous studies have shown conflicting results; Wiersema *et al*. have reported difference in PaO_2_/FiO_2_ ratio after TPE in patients with acute liver failure^12^, however, no difference was found in the study by Larsen *et al*.^11^. Our findings show that ABO-I LDLT recipients have a higher PaCO_2_ level and a higher rate of hypercapnia combined with a lower PaO_2_ level and a higher rate of PaO_2_ ≤ 80 mmHg. Furthermore, based on the previous work^35^ and current results, we believe that a higher rate of PaO_2_ ≤ 80 mmHg can be found in the patients with more severe LC in ABO-I group (Fig. 3). Therefore, for the differential diagnosis of hypoxaemia in LC patients, a TPE procedure a day before surgery should be considered as a possible cause of hypoxaemia.

Although TPE with FFP is known to correct coagulopathies in LC patients^40^, it may unintentionally remove and/or hemodilute platelets and cause platelet dysfunction^21,22^. In line with previous studies^40^, our PSM results showed a slightly improved mean prothrombin time (international normalised ratio, INR) in ABO-I recipients, but there was no difference in CT or CFT in either INTEM or EXTEM. Prospective studies are required to clarify these differences. We found no statistical difference in either the fibrinogen level, the A10 or the MCF in FIBTEM. In contrast, our results showed that platelet counts, A10, and MCF in both INTEM and EXTEM were significantly lower in ABO-I patients, confirming a substantial effect of the reduced platelet counts, which play an important role in haemostasis, particularly in LC^23,41^. Consequently, intraoperative platelet transfusion, a risk factors for mortality after LT^42^, was more frequently required in ABO-I patients.

We found no considerable differences in post-operative outcomes such as duration of intensive care unit stay, 1-month, 6-month and overall mortality and graft failure. Of note, AKIs were significantly more frequent in the ABO-I group. It is possible that inflammatory and allergic responses due to FFP exposure during TPE may play a role in the development of AKI^43,44^; however, this is an area for future research to elucidate the cause of AKI development.

We are aware of the limitations of our study. First, because we used retrospective data analysis, we applied PSM analysis to control for possible selection bias, but effects of residual confounding factors cannot be ruled out. Therefore, a further prospective randomised control study will be necessary to validate our results. Second, our perioperative and TPE protocols and the surgical LT technique may have affected our results because this study was a single-centre study. Therefore, care should be taken when generalising our findings.

In conclusion, in this PSM and IPTW-adjusted analysis, pre-transplant TPE changed the pattern of acid-base balance in LC patients, shifting it from hypocapnic respiratory alkalosis to hypercapnic metabolic alkalosis with hypokalaemia, hypomagnesaemia and hypoxaemia. These altered acid-base and oxygenation statuses should be borne in mind when evaluating ABO-I LDLT recipients, particularly at the initiation of anaesthesia and mechanical ventilation because an unintentional sudden fall in PaCO_2_ may aggravate the electrolyte disturbances leading to severe complications such as severe hypokalaemia. The thrombocytopenia was more prevalent, and a higher rate of platelet transfusions was observed in ABO-I LDLT recipients. We believe that our current result would be informative and lesson to the unexperienced physicians during anaesthesia induction who start the ABO-I LT with pre-transplant TPE. Additionally, whether mitigating the citrate effects of pre-transplant TPE improves outcomes should be further investigated.

## Methods

### Patients

We retrospectively evaluated 2082 consecutive LDLT recipients from November 2008 to May 2015 using a computerised patient data recording system (Asan Biomedical Research Program, ABLE, South Korea). Exclusion criteria were the following: age <18 years (*n* = 123), renal failure (creatinine >1.5 mg/dl) (*n* = 127), lack of preoperative arterial blood gas analysis (*n* = 473) and insufficient data (*n* = 236). In total, 1123 LDLT recipients (923 ABO-C and 200 ABO-I) were included in our final analyses. Of these, 514 LDLT recipients (385 ABO-C and 129 ABO-I) had received rotational thromboelastometry (ROTEM; TEM International GmbH, Munich, Germany) before anaesthesia induction. The recipients’ characteristics, preoperative laboratory and intraoperative transfusion data and ROTEM assay parameters were evaluated and compared. ABO-C LDLT recipients were regarded as typical LC patients as controls to evaluate the effects of TPE in ABO-I LDLT recipients. We used the ascites degree classification by the International Ascites Club^45^ which is graded from zero to five according to the presence of none, mild, moderate, large, diuretics-resistant or diuretics-intractable ascites. All our recipients underwent living donor transplantation according to standardised technique with partial clamping of the inferior vena cava without veno-venous bypass^46^. Methods were carried out in accordance with the relevant guidelines and regulations. The Institutional Review Board (IRB) of Asan Medical Center in Seoul, Korea approved this study [2015–1078] and the requirement for written informed consents was waived by the IRB due to retrospective study design. The Hospital Based Organ Procurement Organization designated by law procured the acquisition of living or deceased liver grafts, and none of the grafts were obtained from executed prisoners. The datasets generated and/or analysed during the current study are available from the corresponding author upon reasonable request.

### Donor selection policy

The detailed donor selection policy and assessments were described elsewhere^47^. In brief, only voluntary donors were evaluated. The first step entailed clinical examinations and serologic tests, the second step included liver computed tomography and abdominal doppler sonography and the last step was a percutaneous liver biopsy. The hepatic steatosis had to be less than 30%, and the left liver volume had to comprise more than 35% of the liver. Legally, donors have to be ≥20 years for organ donation; however, exceptions (≥16 years old) could be applied if the recipients were the parents of donors. ABO-I donors were allocated only if ABO-C donors were unavailable^7^. The ethics committee of the local authority and the Korean Network for Organ Sharing, affiliated with the Korean Ministry of Health, approved every donation.

### Laboratory data

Radial arterial cannulation with local anaesthetic infiltration was performed before initiating anaesthesia to obtain routine blood samples at the Asan Medical Center. Additionally, laboratory data before TPE were obtained from ABO-I LDLT recipients. Alkalosis was defined as pH ≥ 7.45 and severe alkalosis as pH ≥ 7.50 according to the Henderson-Hasselbalch approach, as published^48^. In accordance with the obtained pH values, the metabolic component was evaluated with respect to the bicarbonate level (HCO_3_^−^ > 30 mmEq/l as metabolic excess) and the respiratory component with respect to the PaCO_2_ (> 45 mmHg as hypercapnia)^49^. The PaO_2_ was dichotomised by 80 mmHg for further analysis.

For biochemical data analysis, severe hypokalaemia (≤3.0 mmol/l) and hypomagnesaemia (≤1.7 mg/dl) were defined. We used tCa-to-iCa and serum lactate level as surrogates for blood citrate concentration^4,25,26^; we considered a tCa-to-iCa value > 2.1 as the result of citrate overdosing^25^ and a tCa-to-iCa value > 2.5 as that of a severe citrate accumulation^4,25,26^. Platelet counts were performed on standard EDTA anticoagulated specimen. Thrombocytopenia was considered severe when the platelet count was <30,000/µl. As LC is characterised by deficiency of, both, pro- and anticoagulants^50^, not only conventional laboratory tests but also ROTEM analysis was performed to adequately compare global haemostasis^51^. The tests and nomenclatures used in ROTEM are as follows: (1) INTEM, coagulation activated intrinsically via contact activator; (2) EXTEM, coagulation activated extrinsically via tissue thromboplastin; (3) FIBTEM, coagulation activated as EXTEM with addition of platelet inhibitor; (4) Clotting time (CT); (5) Clot formation time (CFT); (6) A10 (mm), amplitude measured 10 minutes after start of recording; and (7) maximal clot firmness (MCF) (mm), maximal amplitude. Intraoperative transfusions of pRBCs, FFP, cryoprecipitate and apheresis platelets were based on institutional standards adopted from clinical and ROTEM guidelines, which aim to maintain prothrombin time (INR) <2.0, fibrinogen >100 mg/dl and platelet counts >30000/µl, as published^52^.

### Preoperative TPE protocol

The IA titre was measured at the beginning of the evaluation of LT recipients and TPE was repeatedly performed until IA titres dropped below 1:8^53^. IA titres were followed up every day before surgery to ensure a titre <1:8 at the time of anaesthesia induction^14^.

All TPEs were performed with COBE instruments (COBE Spectra; Terumo BCT, CO, USA) using a centrifugal approach via a dual-lumen central venous catheter (MAHURKAR^TM^; Covidien llc, Mansfield, USA) in the internal jugular vein, using AB-type FFP as replacement fluid. The total volume of FFP was calculated to estimate plasma volumes with the following formula: plasma volume = weight (kg) × (1–haematocrit (%)) × (68 ml/kg for men or 62 ml/kg for women)^54^. Anticoagulant Citrate Dextrose Solution A (ACD-A, USP) was infused with whole blood to anticoagulant ratio of 18:1 according to our institution’s protocol. The flow rate was roughly 40 ml/min, adjusted based on patient’s tolerance. Calcium gluconate was infused at a rate of 10–20 mg/h during the entire TPE period, titrated individually for each patient to prevent and treat adverse effects of hypocalcaemia^55,56^.

### Definitions of Outcomes

The patients were followed up until 28 February 2017. Postoperative outcomes included 30-day, 6-month and overall mortality, overall graft failure, intensive care unit stay and AKI development after LT. Mortality data were collected from electronic medical records and the registry, which the Asan Organ Transplantation Center updates regularly. Graft failure was determined if a recipient underwent re-transplantation or died from any cause, whichever was first. Post-operative AKI was defined according to the Kidney Disease Improving Global Outcomes classification, i.e. serum creatinine increases ≥0.3 mg/dL within 2 days after surgery or serum creatinine increases ≥1.5-fold within 7 days after surgery.

### Statistical analysis

Continuous variables are presented as mean ± standard deviation or median and interquartile ranges, according to normality. Categorical variables are presented as percentages and frequencies. Between-group comparisons were evaluated using a Student’s *t*-test, Mann–Whitney *U*-test, chi-square or Fisher exact tests, as appropriate. Comparisons between pre- and post-TPE in ABO-I patients were done with a paired t-test, Wilcoxon signed rank test or McNemar test, as appropriate. To assess the effect of LC severity on the clinical variables, the MELD score was divided into tertiles (≤10, 11–20, >20). The linear-by-linear chi-square test, Jonckheere-Terpstra test for trend analysis, and analysis of covariance test were applied as appropriate. Logistic regression and Cox proportional hazards regression analyses were performed to analyse the outcomes.

To minimise intergroup differences in baseline characteristics, a 1:1 PS was calculated using greedy matching algorithms by clinically relevant covariates shown in Table 1 without regards to outcomes. For a smooth PSM analysis, we converted the MELD score and the aspartate aminotransferase and alanine aminotransferase measurements into categorical values by using the cut-off values of 15, 40 and 40, respectively. We conducted a separate PSM analysis in 514 patients who underwent ROTEM analysis. Model discrimination and calibration were evaluated with c-statistics and Hosmer-Lemeshow statistics, respectively. After matching, a paired *t*-test or Wilcoxon signed rank test for continuous variables and McNemar test for categorical variables were used. Logistic regression analyses with generalised estimating equations and a logit link were used to compare the risk of each outcome in the PSM cohort. Furthermore, we performed IPTW to stringently adjust for significantly different characteristics and confounding factors between the patients, as previously performed^57^. All *P* values < 0.05 were considered statistically significant. The R software version 3.3.2 or SAS® version 9.4 (SAS Institute, Cary, NC, USA) were used for data manipulation and statistical analyses.

## Electronic supplementary material

Supplementary Table S1 and S2
