# Supplementary material for: Prevalent metabolic derangement and severe thrombocytopenia in ABO-incompatible liver recipients with pre-transplant plasma exchange
Source: Sci Rep. 2018 Apr 27;8:6679. doi: 10.1038/s41598-018-24887-x (PMC5923210; doi:10.1038/s41598-018-24887-x)
Supplement: Supplementary file 1 — Supplementary Table S1 and S2 [file 41598_2018_24887_MOESM1_ESM.docx]

**Prevalent metabolic derangement and severe thrombocytopenia in ABO-incompatible liver recipients with pre-transplant plasma exchange**

Hye-Mee Kwon M.D.^1^, In-Gu Jun Ph.D.^1,*^, Jung Bok Lee M.D., Ph.D.^2^, Young-Jin Moon M.D.^1^, Kyeo-Woon Jung M.D.^1^, Hye-Won Jeong M.D.^1^, Yong-Seok Park M.D.^1^, Jun-Gol Song M.D., Ph.D.^1^, and Gyu-Sam Hwang M.D., Ph.D.^1^

^1^Department of Anesthesiology and Pain Medicine, Laboratory for Cardiovascular Dynamics, Asan Medical Center, University of Ulsan College of Medicine, 88 Olympic-ro 43-gil, Songpa-gu, Seoul, 05505, Republic of Korea

^2^Department of Clinical Epidemiology and Biostatistics, University of Ulsan College of Medicine, Asan Medical Center, Seoul, 88 Olympic-ro 43-gil, Songpa-gu, Seoul, 05505, Republic of Korea

**Supplementary Table S1.** Laboratory results of acid-base balance, electrolytes, respiratory analyses, blood counts and coagulation parameters in ABO-incompatible liver transplantation recipients before therapeutic plasma exchange (TPE) and after TPE, obtained immediately before surgery.

|  | Before TPE | After TPE | *P* value |
| --- | --- | --- | --- |
| **Acid-base balance (n = 94)** |  |  |  |
| pH | 7.45 ± 0.03 | 7.47 ± 0.04 | <0.001 |
| pH ≥ 7.50, % | 6 (6.4) | 24 (25.5) | <0.001 |
| Base excess, mmEq/l | −2.22 ± 3.04 | 5.13 ± 3.86 | <0.001 |
| HCO_3_^-^, mmEq/l | 22.0 ± 3.0 | 29.2 ± 3.9 | <0.001 |
| HCO_3_^-^ > 30 mmEq/l, % | 1 (1.1) | 31 (33) | <0.001 |
| PaCO_2_, mmHg | 31.2 ± 4.5 | 39.0 ± 4.7 | <0.001 |
| PaCO_2_ ≥ 45 mmHg, % | 0 (0) | 10 (10.6) | 0.001 |
| PaO_2,_ mmHg | 131.8 ± 33.8 | 84.3 ± 11.2 | <0.001 |
| PaO_2_ ≤ 80 mmHg | 4 (4.3) | 37 (39.4) | <0.001 |
| Lactic acid, mmol/l | 2.14 ± 1.35 | 1.07 ± 0.36 | 0.011 |
| **Electrolyte analysis (n = 200)** |  |  |  |
| Sodium, mmol/l | 137 ± 4.9 | 138 ± 3.4 | <0.001 |
| Potassium, mmol/l | 4.1 ± 0.5 | 3.4 ± 0.45 | <0.001 |
| Potassium ≤ 3 mmol/l, % | 2 (1.0) | 36 (18.0) | <0.001 |
| Magnesium, mg/dl, n=54 | 1.94 ± 0.27 | 1.87 ± 0.26 | 0.062 |
| Total calcium, mmol/l | 2.06 ± 0.14 | 2.07 ± 0.18 | 0.780 |
| Ionised calcium, mmol/l | 1.20 (1.15–1.25) | 1.12 ± 0.07 | <0.001 |
| Ratio of total to ionised calcium > 2.1, % | 5 (2.5) | 18 (9.0) | 0.004 |
| **Coagulation parameters (n = 200)** | | | |
| Platelets, ×10^3^ /µl | 58 (39–92) | 47 (32–72) | <0.001 |
| Platelets < 30x10^3^ /µl | 29 (14.5) | 38 (19.1) | 0.003 |
| Fibrinogen, mg/dl | 159 (130–205) | 165(145–193) | <0.001 |
| Antithrombin III, % | 48 (36–67) | 61 (50–75) | <0.001 |
| PT, INR | 1.38 (1.18–1.60) | 1.25 (1.14–1.42) | <0.001 |
| aPTT, s | 33 (30–38) | 31 (28–34) | <0.001 |

*Values are expressed as mean (±standard deviation), or median and interquartile range for continuous variables, as appropriate, and n (%) for categorical variables. aPTT, activated partial thromboplastin time; INR, international normalised ratio; PT, prothrombin time; TPE, Therapeutic plasma exchange.

**Supplementary Table S2.** Patient characteristics and laboratory data of ABO-C and ABO-I liver transplantation recipients who underwent rotatory thromboelastometry, before and after propensity score matching.

|  |  | **Total set** | | | **Propensity matched set** | | |
| --- | --- | --- | --- | --- | --- | --- | --- |
|  | ABO-C  (n = 385) | ABO-I  (n = 129) | *P* value | ABO-C  (n = 129) | ABO-I  (n = 129) | P value | Standardised  mean difference |
| **Patient demographics** |  |  |  |  |  |  |  |
| Gender, male, % | 297 (77.1) | 93 (72.1) | 0.298 | 99 (76.7) | 93 (72.1) | 0.476 | 0.107 |
| Age, years | 52.9 ± 8.7 | 53.3 ± 8.2 | 0.650 | 53.4 ± 8.7 | 53.3 ± 8.2 | 0.912 | 0.014 |
| Body mass index, kg/m^2^ | 23.8 ± 3.3 | 23.4 ± 2.9 | 0.283 | 23.3 ± 3.2 | 23.4 ± 2.9 | 0.686 | 0.050 |
| MELD score | 13.7 ± 6.9 | 11.5 ± 4.5 | <0.001 | 11.4 ± 4.8 | 11.5 ± 4.5 | 0.841 | 0.025 |
| MELD ≥ 15, % | 131 (34.0) | 29 (22.5) | 0.019 | 32 (24.8) | 29 (22.5) | 0.769 | 0.055 |
| Ventilatory care, % | 5 (1.3) | 0 (0) | 0.434 | 0 (0) | 0 (0) | 1.000 | <0.001 |
| **Comorbidities** |  |  |  |  |  |  |  |
| Diabetes, % | 98 (25.5) | 21 (16.3) | 0.044 | 21 (16.3) | 21 (16.3) | 1.000 | <0.001 |
| Hypertension, % | 57 (14.8) | 16 (12.4) | 0.596 | 18 (14.0) | 16 (12.4) | 0.854 | 0.046 |
| Coronary arterial disease, % | 28 (7.3) | 13 (10.1) | 0.407 | 10 (7.8) | 13 (10.1) | 0.662 | 0.082 |
| Hepatic encephalopathy, % | 49 (12.7) | 7 (5.4) | 0.032 | 6 (4.7) | 7 (5.4) | 1.000 | 0.035 |
| Ascites, grades | 2.0 ± 1.0 | 2.0 ± 1.0 | 0.920 | 2.0 ± 1.1 | 2.0 ± 1.0 | 0.680 | 0.055 |
| Current use of beta blocker, % | 73 (19.0) | 32 (24.8) | 0.194 | 35 (27.1) | 32 (24.8) | 0.776 | 0.053 |
| Current use of diuretic, % | 140 (36.4) | 54 (41.9) | 0.313 | 53 (41.1) | 54 (41.9) | 1.000 | 0.016 |
| **Pre-operative variables** |  |  |  |  |  |  |  |
| Total bilirubin, mg/dl | 5.0 ± 8.1 | 2.6 ± 3.6 | <0.001 | 2.3 ± 3.4 | 2.6 ± 3.6 | 0.440 | 0.096 |
| Creatinine, mg/dl | 0.7 ± 0.2 | 0.7 ± 0.2 | 0.005 | 0.7 ± 0.2 | 0.7 ± 0.2 | 0.428 | 0.099 |
| Aspartate aminotransferase > 40 IU/l^,^ % | 87 (22.6) | 14 (10.9) | < 0.001 | 17 (13.2) | 14 (10.9) | 0.702 | 0.072 |
| Alanine aminotransferase > 40 IU/l, % | 188 (48.8) | 35 (27.1) | < 0.001 | 35 (27.1) | 35 (27.1) | 1.000 | <0.001 |
| **Original liver disease, %** |  |  |  |  |  |  |  |
| Hepatitis B virus | 248 (64.4) | 88 (68.2) | 0.497 | 90 (69.8) | 88 (68.2) | 0.893 | 0.034 |
| Hepatitis C virus | 42 (10.9) | 12 (9.3) | 0.727 | 14 (10.9) | 12 (9.3) | 0.836 | 0.052 |
| Alcoholic cirrhosis | 63 (16.4) | 17 (13.2) | 0.469 | 21 (16.3) | 17 (13.2) | 0.598 | 0.088 |
| Other disease | 43 (11.2) | 17 (13.2) | 0.648 | 8 (6.2) | 17 (13.2) | 0.092 | 0.238 |

*Values are expressed as mean (±standard deviation), or median and interquartile range for continuous variables, as appropriate, and n (%) for categorical variables. PS model was discriminated with c-statistics (C = 0.6861) and model calibration was performed with Hosmer-Lemeshow statistics (χ2 = 8.0307, degrees of freedom, DF = 8, p = 0.431). ABO-C, ABO-compatible; ABO-I, ABO-incompatible; MELD, Model for End-stage Liver Disease.
